# Dangers of Antipyrine

**Published:** 1888-08

**Authors:** F. A. Harrington


### Translations.

### DANGERS OF ANTIPYRINE.

Translated from *Les Progress Medicale*,

By F. A. HARRINGTON, A. M., M. D.

Language is the best and the worst of things, quoth Æsop, so sometimes a medicine, extolled very highly as a panacea by some, is brought before a medical tribunal by some judge, who attributes to it a number of misdeeds. And then many accusers appear, and they have a trial of the therapeutic agent, which, if the accusers are credited, is proven to be the cause of the evil Such has been the history of antipyrine. At first only encomiums were showered on that marvelous medicine. We are aware in how many instances it is employed: to reduce febrile temperature, to soothe migraine and the suffering of various nervous diseases, to obviate seasickness, *etc.;* it has been suggested to use it as an antiseptic, an anti-hemorrhagic, etc., etc.

Although antipyrine is so valued, and its efficacy so lauded, still we notice that in certain cases it produces phenomena, if not serious, at least unpleasant and annoying to the physician, which may be styled a sort of antipyrine intoxication. Prof. Ball, under the pseudonym of Dr. O. Jennings, has just related before the Academy of Medicine some of the unpleasant symptoms (fourteenth of February, 1888). Prof. Peter next, in a clinic on refrigerants in typhoid fever, directed his attack against antipyrine, and, at the same time, against other internal antipyretics used universally in our hospitals.

Last year, in reviewing the department of therapeutics, I described in the JOURNAL (seventeenth December, 1887,) some unpleasant phenomena in the use of antipyrine, similar to the following related by Drs. Torsbrook and Whitehouse, and observed in girls and young women. They were gastric pain, fright, screaming, writhing, and an urticarial eruption, accompanied by itching. In one instance there was loss of consciousness.

Dr. Ball's case was a woman sixty-seven years old, who began on the twenty-third of January to take a dose of 2.50 grams of antipyrine, and continued such treatment for eight days for the relief of an attack of rheumatism. At the termination of that time her face became red and swollen, her eyelids were œdematous and nearly closed. Soon there was a rash that extended to the limbs, presenting some garnet-colored patches, almost confluent; a slight pruritus existed. On the succeeding day catarrhal conjunctivitis was present; the eruption still persisted, and the pulse, whose normal rate was thirty-five in the case of the old woman, bounded up to seventy. There was a slight fall of temperature, the patient experienced chilly sensations and *tinnitus aurium*. After the exhibition of a small dose of the tincture of belladonna, the eruption disappeared, but prostration remained. Dr. O. Jennings has found similar cases cited by Allen Sturge, Whitehouse and Barbe, following the administration of antipyrine in doses of 0.25 centigrams to one gram. All the symptoms mentioned above resemble somewhat an attack of urticaria after the ingestion of shellfish; only the severe itching and the dyspnœa are absent. Hardy has given an account of a parallel case which had severe itching (Academy of Sciences, February 21st); also Mayet, one to the Therapeutic Society (March 4th). In the latter the urticaria appeared after three grams of antipyrine had been taken, and afterwards, a dose of 0.50 centigrams having been given, an œdema of eyelids and face appeared. In the experience of Prof. G. See, who has met similar cases, these symptoms are rare and not so grave as commonly believed. It may happen that a patient who has taken a dose of three to four grams of antipyrine for one or two weeks, may show an eruption, urticarial or not, which disappears after a day or two. In men, G. See noted it in one in fifty cases or thereabouts; it is oftener met with in women, one in twelve or fifteen cases. Darenburg has observed this rash particularly in tuberculosis, but without evil consequence, after treatment with antipyrine; but these phenomena are absolutely painless, and in such cases without any intoxication, so to speak. The facts related above are exceptional. The cutaneous eruption is analogous to that after the ingestion of quinine or iodide of potassium. There is, after a time, an intolerance of antipyrine, and a dose, however small, causes an eruption; then it should be discontinued. Acetanilide will then replace it with advantage.

Besides the dermatological manifestation, other phenomena are observed, some serious, as are those described by Peter at his clinic (*Bulletin Medical*, April 25th). In the first case, a patient fifty-two years old, with typhoid fever, epistaxis supervened after the continuous administration of antipyrine for a fortnight, and the man died of a cachectic purpura and debility. The second case is a woman, twenty-six years old, also afflicted with typhoid fever, in whom antipyrine had caused uræmic symptoms and ultimately eclampsia and death. We might be justified in the belief that those symptoms would have occurred in these typhoid patients if they had not been treated with antipyrine—in the first, because there was an ulceration of Peyer's patches of a hemorrhagic type; in the second, because the kidneys were involved in the general infection.

Finally, some phenomena more benign have been recorded: emesis, gastric disturbances and anorexia when the medication is prolonged. Hardy has mentioned cerebral depression, cardiac disorders, syncope, and an instance of amnesia lasting eighteen hours. In addition, Dr. Queirel cites a case of chronic gastritis with trigeminal neuralgia, in which the gastric symptoms were relieved, but the neuralgia persisted. Antipyrine in doses of 0.50 centigrams was prescribed hypodermically for the woman, but the gastric trouble did not recur.

Other writers have noticed coldness of the extremities; it should be remarked, however, that this was in old people, in whom cold feet are common. Antipyrine, as we well know, has also been recommended for parturient women, particularly at the time of labor.

Dr. Chéron saw some unpleasant symptoms developed in a pregnant woman suffering with typhoid fever, when antipyrine was given her: subnormal temperature, cramps, difficulty of speech, impairment of hearing, and dullness of intellect. He attributed these to the pregnant condition of the woman. On the contrary, Dr. Queirel has used antipyrine advantageously in all the periods of pregnancy. It has no unfavorable influence on the course of the labor, hastens the period of dilatation, and facilitates expulsion, which becomes nearly painless. He says its employment is not contra-indicated.

Laborde, in explaining before the Academy of Sciences the physiological effects of antipyrine upon the nervous system, showed it diminished sensation and the reflexes.

An exaggeration of these phenomena would produce symptoms referable to the brain; the eruption would be due to vaso-motor disturbances of the same origin. Such symptoms would then be seen, particularly in extremely excitable and nervous persons, and, in point of fact, we see that women present them oftenest. The majority of infants, however, according to Allivier (Academy of Medicine), bear perfectly well a dose of three or four grams of antipyrine; nevertheless, a few have an eruption. This is a remarkable idiosyncrasy. On the other hand, Lepine and Porteret have shown by experiment that antipyrine increases the glycogenic function of the liver, and so arrests the transformation of glycogen into sugar. In proof of that discovery, Hichard and Dujardin-Beaumetz have recognized the value of the drug in polyuria, and in diabetes mellitus. From a study of these facts, we see that antipyrine diminishes the amount of urine; if, therefore, it be given to patients with renal trouble, the excretion by the kidney of the products of metamorphosis becomes more difficult. So antipyrine might produce uræmic intoxication; you can thus explain the facts related by Prof. Peter. It is, consequently, a necessity, as Dr. Reliquet says, to make a urinary analysis before prescribing antipyrine.

You might, too, attribute the untoward symptoms that have been named to an impurity of antipyrine, which has been carelessly manufactured, and contains benzin. Prof. G. See has actually associated with the drug bicarbonate of soda or Seltzer water, to obviate its inconveniences. Subcutaneous injections are also productive of mischief. Prof. Ball has recommended tincture of belladonna in cases of urticaria. A sub-normal temperature yields usually to the means ordinarily employed to restore warmth.

It is to be hoped that the French manufacturers will supply us with purer samples of analgesine or dimethyloxyquinizine. The Academy has been annoyed by numerous pharmacists dealing out antipyrine as so much licorice to their patrons, without prescriptions, at every turn, and to anyone who complained of any pain whatsoever. Here is a wrong which it is necessary to right at any price. The pharmacist should not hand out this drug indiscriminately.

We have reviewed all the charges against antipyrine; we are, nevertheless, justified in saying it is a good drug, certain in its action; a drug which should be handled with circumspection, and whose use will be more restrained when we better understand its effects, its dose, and occasion for its exhibition. We shall then have no more of those unfortunate symptoms, particularly if we can be absolutely certain of its purity.

